# Prospective associations between hand grip strength and subsequent depressive symptoms in men and women aged 50 years and older: insights from the Survey of Health, Aging, and Retirement in Europe

**DOI:** 10.3389/fmed.2023.1260371

**Published:** 2023-09-14

**Authors:** Sarah Mendorf, Aline Schönenberg, Konstantin G. Heimrich, Tino Prell

**Affiliations:** ^1^Department of Neurology, Jena University Hospital, Jena, Germany; ^2^Department of Geriatrics, Halle University Hospital, Halle, Germany; ^3^Department of Geriatrics, Jena University Hospital, Jena, Germany

**Keywords:** depression, depressive symptoms, general health, older adults, hand grip strength, SHARE

## Abstract

**Introduction:**

In previous cross-sectional and longitudinal studies, depressive symptoms have been associated with lower hand grip strength (HGS), which is a convenient measure of overall muscular strength and serves as a marker of poor health. Most studies have considered low sample sizes or highly selective patient cohorts.

**Methods:**

We studied the association between depressive symptoms (EURO-D) and HGS in three waves from the cross-national panel dataset Survey of Health, Aging, and Retirement in Europe (SHARE). Linear regressions and Generalized Estimating Equations (GEE) were conducted to determine factors associated with depressive symptoms and investigate whether HGS predicts future depressive symptoms.

**Results:**

Cross-sectional HGS explained 7.0% (Wave 4), 5.7% (Wave 5), and 6.4% (Wave 6) of the EURO-D variance. In the GEE, we analyzed people without depression in Wave 4 (*N* = 39,572). HGS predicted future EURO-D (*B* = −0.21, OR = 0.979, 95%CI (0.979, 0.980), *p* < 0.001) and remained a significant predictor of future depressive symptoms after adjustment for age, sex, psychosocial and physical covariates.

**Discussion:**

Muscle strength is a known marker for physical health, but a relation with mental health has also been proposed previously. This study confirmed the link between HGS and depressive symptoms in men and women aged ≥50 years in a large longitudinal dataset. Further research is required to understand the mechanisms behind this link to determine whether HGS can serve as a specific marker of depressive symptomology, or whether they coexist due to common underlying disease processes.

## 1. Introduction

Depression is a significant global health concern, affecting millions of people worldwide (1). People with depression are at increased risk for poor quality of life (QoL), anxiety, loneliness, poor sleeping quality (2), and several somatic comorbidities (3–6). There is growing evidence that physical fitness is an important modifying factor for depression (7, 8). One component of physical fitness is muscular strength. A reliable and convenient measure of an individual’s muscular strength is hand grip strength (HGS) when measured in standard conditions. It is assessed using hand dynamometers, which are safe, cost-effective, and easy to use (9, 10). Several studies with differing methodological approaches have attempted to understand how HGS is not only associate with physical, but also mental health. For example, several cross-sectional studies have found that individuals with lower HGS have a higher prevalence of depressive symptoms (11–13) while others did not (14–17). However, cross-sectional results cannot provide information on causality, therefore, research has also investigated the relationship between HGS and the development of depressive symptoms over time. Overall, a recent meta-analysis summarizing prospective cohort studies found that higher HGS was related to a decreased risk of depressive symptoms with a pooled risk ratio of 0.74 (95% confidence intervals (CI) 0.65–0.85) in males (18). However, several questions remain. As an unspecific marker for general health, HGS is associated with a plethora of age-related disorders and functional limitations (19–22). Therefore, only analyses with adjustment for various cofactors – especially psychosocial factors – are suitable to understand the relationship between HGS and depression. Moreover, many prospective studies, although longitudinal, only considered one follow-up investigation of depression (23–29), making it difficult to differentiate between actual development and measurement errors. Some studies applied more than two measurement points in an attempt to accurately depict an individual’s development, however, these studies considered highly selective patient groups or have small sample size (16, 30). For example, Van Milligan and colleagues (31) assessed only people up to the age of 65, while Stessmann et al. only included persons between the ages of 70 and 90 (15). Gariballa et al. only assessed their participants over a time-span of 6 months (32). Thus, while some evidence points toward a relation between HGS and depressive symptomology, this evidence is not sound and requires further investigation using longitudinal, multi-timepoint data with sufficient sample sizes.

We therefore conducted this analysis of longitudinal measurements using the Survey of Health, Aging and Retirement in Europe (SHARE). This is a large multi-national panel dataset of more than 140,000 subjects, aged 50 and over, from 20 European countries and Israel. SHARE is an ongoing research collaboration to study the impact of health, social, economic, and environmental policies throughout the life of middle-aged and older people in Europe. At the present time, 8 waves of SHARE data have been collected with repeating and new participants, allowing for cross-sectional and longitudinal data analysis of a broad variable spectrum. These variables are collected by trained staff in each country using a computer-assisted personal interviewing procedure, sometimes supplemented by drop-off paper-pencil questionnaires. In addition, participants were invited into study centers to complete health assessments such as cognitive functioning, grip strength, walking speed or blood samples (33).

## 2. Methods

### 2.1. Study design and population

The data for this study was collected by the respective country teams of the SHARE survey. The methods used in the survey, the sampling design, and the data resources were all described previously in detail (34–39). Probabilistic sampling was used to make sure that the participants chosen were nationally representative. The households that were selected had to include at least one person aged 50 or older, who was chosen as the primary respondent. The questions were asked using a Computer Assisted Personal Interview (CAPI) and a paper and pencil questionnaire. The survey questions were related to the demographic, socio-relational, and health-related (including functional ability and mental health) measures. The data used in this study is taken from SHARE waves 4 (2011) to 6 (2015) (40–42).

As the main variable of interest in our analysis was depressive symptomology, we excluded the participants who did not complete the questionnaire depicting depressive symptoms. This leads to 58,000 participants in wave 4, 66,065 in wave 5, and 68,085 in wave 6. Detailed information on this sample is provided in Table 1. For the longitudinal analysis, we used HGS in wave 4 as a predictor for future depressive symptoms. Here we analyzed people without clinically relevant depressive symptoms (EURO-D < 4) at baseline in wave 4 who also participated in the two subsequent waves (*N* = 39,572).

**Table 1:** Descriptive statistics.

		W4 (*N* = 58,000)		W5 (*N* = 66,065)		W6 (*N* = 68,085)	
Categorical variables		*n*	%	*n*	%	*n*	%
Sex	Male	25,087	43.3	29,114	44.1	29,663	43.6
	Female	32,913	56.7	36,951	55.9	38,422	56.4
Living situation	Living with a spouse/partner in household	42,122	72.6	48,491	73.4	49,122	72.1
	Living without a spouse/partner in household	15,878	27.4	17,574	26.6	18,963	27.9
Body mass index categories (43)	Below 18.5-underweight	722	1.3	851	1.3	792	1.2
	18.5–24.9 – normal	19,800	36.1	23,702	36.9	23,281	35.1
	25–29.9 – overweight	22,463	40.9	26,213	40.8	27,484	41.5
	30 and above – obese	11,924	21.7	13,433	20.9	14,732	22.2
Smoke at present time	Yes	10,753	18.7	11,636	17.6	2,794	6.5
	No	46,703	81.3	54,313	82.4	40,075	93.5
Days a week consumed alcohol in the last 6 months	Not at all	18,601	32.5	21,305	32.3	0	0
	Less than once a month	6,284	11.0	6,319	9.6	0	0
	Once or twice a month	6,796	11.9	7,880	12.0	0	0
	Once or twice a week	9,749	17.0	12,388	18.8	0	0
	3 or 4 days a week	3,774	6.6	4,811	7.3	0	0
	5 or 6 days a week	1,617	2.8	1910	2.9	0	0
	Almost every day	10,454	18.3	11,261	17.1	0	0
Finances (Is household able to make ends meet?)	With great difficulty	6,521	11.5	5,555	8.6	8,317	12.6
	With some difficulty	16,443	29.0	15,478	24.1	17,625	26.6
	Fairly easily	18,583	32.7	19,042	29.6	17,750	26.8
	Easily	15,206	26.8	24,267	37.7	22,459	34.0

Metric variables	*m*	SD	*m*	SD	*m*	SD
EURO-D	2.59	2.30	2.39	2.25	2.45	2.28
Age at interview (in years)	65.91	10.37	66.77	10.33	67.63	10.30
Years of education (in years)	10.56	4.31	11.15	4.34	10.88	4.41
Self-rated health (SRH)*	3.25	1.08	3.14	1.09	3.19	1.07
Number of chronic diseases	1.23	1.27	1.18	1.24	1.20	1.25
Hand grip strength (HGS)	33.81	11.98	33.70	11.83	33.18	11.76
Activities of daily living (ADL)*	0.24	0.77	0.24	0.80	0.25	0.82
Instrumental activities of daily living (IADL)*	0.20	0.75	0.22	0.81	0.23	0.82
Mobility index*	0.57	0.97	0.54	0.98	0.58	0.99
Body mass index (BMI)	26.88	4.73	26.82	4.73	26.99	4.62

*higher values indicate difficulties (ADL, IADL, mobility) or poorer SRH.

### 2.2. Measures

#### 2.2.1. Dependent variable

Depressive symptoms were measured with the EURO-D scale (44). The EURO-D scale was designed to compare depressive symptomology in seniors from 11 nations in Europe. This scale consists of 12 dichotomous items that refer to the following symptoms: sadness, pessimism, suicidality, guilt, sleep problems, interest in things, irritability, poor appetite, fatigue, difficulty concentrating, enjoyment, and tearfulness. Items are scored 0 or 1 such that 1 always indicates negative valence (i.e., 1 = more depressed) and summed for a final score between 0 and 12, where a summary score ≥ 4 indicates clinically relevant depressive symptomology (44).

#### 2.2.2. Independent variables

Variables were obtained from easySHARE (45) which have been carefully documented by SHARE online and with corresponding PDF codebooks.^1^

HGS was measured via hand dynamometer. Two measurements were taken on each hand, alternating between the hands. The highest value was used for further analysis. According to the Share Manual (45), HGS is recorded as the maximum value of grip strength measurements of both hands. For this purpose, the dynamometer handle is adjusted to fit the hand of the respective participant, with the handle resting comfortably against the middle part of the index finger. If possible, the assessment is performed standing up with rings removed. Each hand is assessed twice in alternating order. Study staff is systematically instructed and trained to perform the HGS assessments as harmoniously as possible across countries.

We used HGS as metric variable and additionally we divided HGS into low and normal HGS according to the thresholds provided by Cruz-Jentoft et al. (46). Low HGS was defined when the HGS measurement fell below 27 kg (men) or below 16 kg (women). The use of cut-off points for HGS is helpful for clinical practice where they inform about the need for intervention, and oftentimes, studies report both metric and categorical information (47).

Furthermore, in addition to common sociodemographic variables such as marital state and education, we selected factors already described in the literature that influence depression, including age and gender (48), body mass and activities (49), functional status (47), substance consumption (50), and overall health (51). As financial situation is closely linked to body mass, health, and family burden, which is also related to depressive symptoms, we additionally included it (51, 52). For ease of reproducibility, the italics indicate the variables as they are named in the SHARE dataset (45): sex (female = 1, male = 0), age at interview (years), years of education, living situation (living with a spouse/partner in household or not, *partnerinhh*), self-perceived/rated health (SRH, *sphus*), number of chronic diseases (*chronic_mod*), activities of daily living/ADL (*adla*, high values indicates difficulties in ADL, range 0–5), instrumental ADL index/IADL (*iadlza*, high values indicates difficulties in IADL, range 0–5), mobility index (*mobility,* high values indicate mobility difficulties), body mass index (BMI), smoking at present time (*smoking*), alcohol consumption (*br01_mod*, Days a week consumed alcohol last 6 months. Answers: 1 – ‘not all all’ to 7 – ‘almost every day’), finances (co007_, Is household able to make ends meet?; Answers: 1 – ‘With great difficulty’, 2 – ‘With some difficulty’, 3 – ‘Fairly easy’, 4 – ‘Easily’).

SRH was assessed by asking participants how they rated their health in general using a 5-point Likert scale (excellent, very good, good, fair, and poor). For ADL the following ADLs were asked about and summed up: dressing, bathing or showering, eating, cutting up food, walking across a room, and getting in or out of bed. For IADL the following IADLs were asked about and summed up: telephone calls, taking medications, managing money, shopping for groceries, and preparing a hot meal. Mobility index was summed up from walking 100 meters, walking across a room, climbing several flights of stairs, and climbing one flight of stairs. The higher the index, the lower the mobility of the respondent (range 0–4).

## 3. Statistical analysis

All analyses were conducted using IBM SPSS statistics (Version 27). Descriptive statistic was used to characterize the cohorts. Values are given as mean with standard deviation (SD) or numbers and percentages. The included parameters were normally distributed, as assessed by the Shapiro–Wilk-Test, *p* < 0.05. Missing data were treated according to the pairwise deletion process. Linear regression analyses with stepwise selection and Akaike information criterion (AIC) as selection criterion were used to determine the factors that are associated with EURO-D in each wave. Given that multicollinearity was observed between SRH and mobility index, only SRH was entered into the regression models as it was previously identified to influence depressive symptoms (51), and it contains more encompassing health information than solely mobility. In addition, finances, ADL, IADL, sex, number of chronic diseases, age, HGS, living situation, BMI, years of education, smoking, and alcohol consumption were initially added to all models. Generalized Estimating Equation (GEE) were used to investigate a possible longitudinal effect of HGS on depressive symptoms (EURO-D). GEE is a ‘marginal’ longitudinal method that estimates mean relationships and deals with nuisance covariances separately to get a better estimate and valid significance tests (53). GEE estimates two equations, one for mean relations and one for covariance structure. As the use of GEE requires for the specification of the underlying data structure, we tested the model with Gaussian and Poisson distribution as well as different correlation structure (independent, exchangeable) (54). The best model fit based on the Quasi-likelihood under independence model criterion (QIC) (55) was reached for a poisson distribution and independent correlation structure for EURO-D. The variables that proved to be significant in linear regression were chosen as covariates.

## 4. Results

Descriptive statistics of the three waves are summarized in Table 1. According to the criteria described by Cruz-Jentoft et al. (46), HGS was low in 6% (wave 4 and 5) to 6.4% (wave 6) of participants. Using a EURO-D summary score ≥ 4 (44), depression was present in 28.5% subjects in wave 4, 25.2% in wave 5, and 26% in wave 6.

### 4.1. Cross-sectional analysis

**Figure 1:**
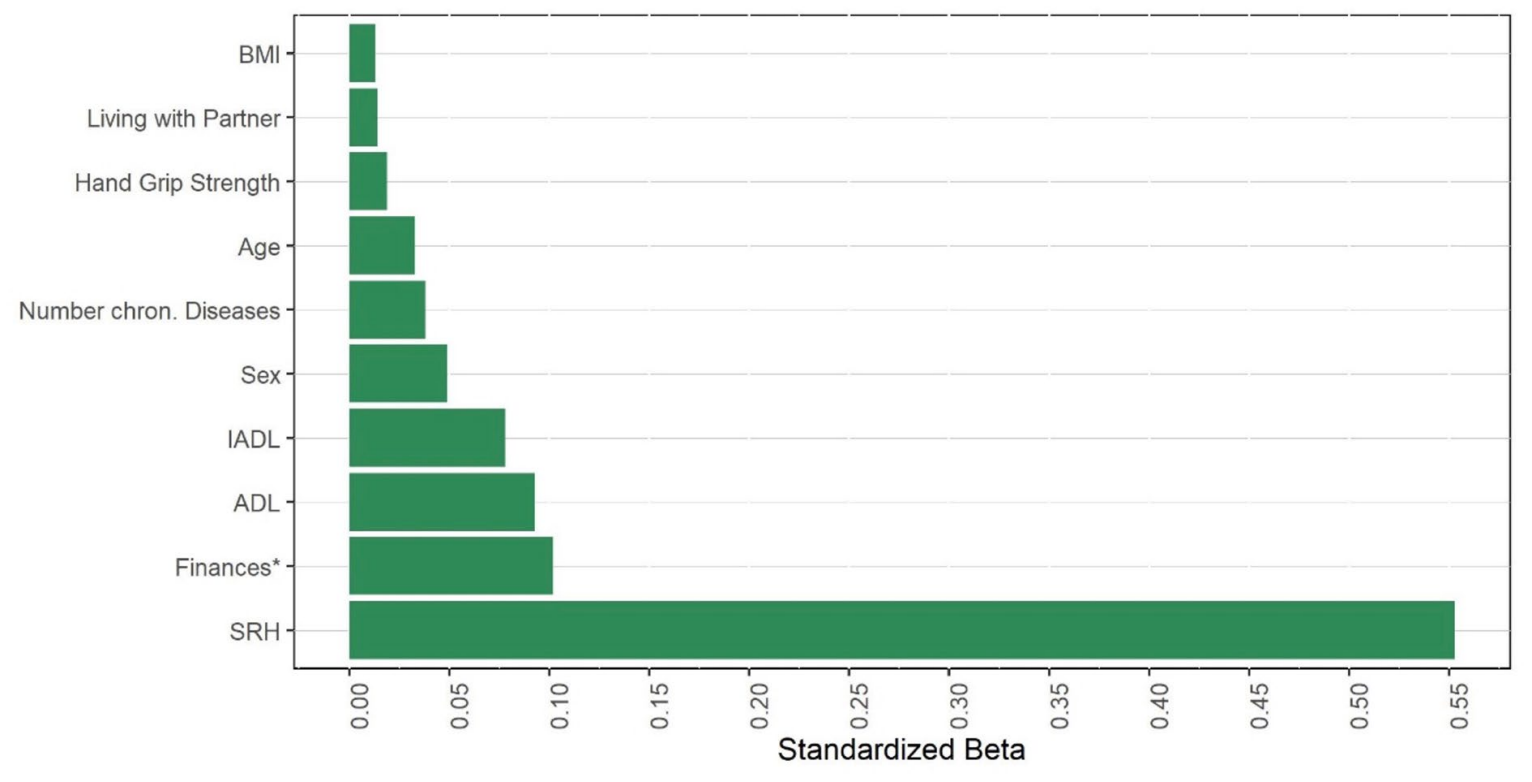
Factors associated with EURO-D (cross-section in wave 4). Finances refers to the item. Is household able to make ends meet? SRH, self rated health. (I)ADL, (instrumental) activities of daily living. BMI, body mass index.

First, we determined the cross-sectional association between HGS and depressive symptoms. The variance of the EURO-D that can be explained HGS alone ranged between 7.0% (wave 4), 5.7% (wave 5), and 6.4% (wave 6). Also, with adjustment for various covariates, HGS was significantly associated with the EURO-D in each wave (wave 4: adjusted *R*^2^ = 0.283, *p* < 0.001; wave 5: adjusted *R*^2^ = 0.283, *p* < 0.001; wave 6: adjusted *R*^2^ = 0.270, *p* < 0.001). The strongest association with depressive symptomology were found for poorer, worse economic situation, and more difficulties in ADL (Figure 1; Supplementary Tables S1–S3).

### 4.2. Longitudinal analysis

Next, we determined if HGS can predict future depressive symptoms. To assess its predictive value, we calculated several GEE models with the EURO-D as dependent variable using HGS in wave 4 as a predictor. Here we analyzed people without clinically relevant depressive symptoms (EURO-D < 4) at baseline in wave 4 (*N* = 39,572). In the GEE without adjustment for covariates, HGS significantly predicted future EURO-D (*B* = −0.21 OR = 0.979, 95%CI [0.979, 0.980] *p* < 0.001). HGS remained a significant predictor of future depressive symptoms also with adjustment for age, sex, and various psychosocial and physical covariates that were cross-sectionally associated with depressive symptomology (Table 2). However, the predictive influence of HGS is low according to the OR of almost 1.

**Table 2:** GEE with adjustment for various covariates: dependent variable EURO-D.

	*B*	Standard error	*p*	OR	95%CI lower	95%CI upper
Constant	0.509	0.0302	<0.001	1.664	1.568	1.765
Age	−0.005	0.0003	<0.001	0.995	0.994	0.995
Hand grip strength	−0.008	0.0003	<0.001	0.992	0.991	0.993
Sex (male)	−0.169	0.0075	<0.001	0.845	0.832	0.857
Living with spouse/partner (yes)	−0.068	0.0059	<0.001	0.935	0.924	0.945
Is household able to make ends meet (with great difficulties)	0.315	0.0082	<0.001	1.370	1.348	1.392
Is household able to make ends meet (with some difficulties)	0.115	0.0064	<0.001	1.122	1.108	1.136
Is household able to make ends meet (fairly easy)	0.003	0.0060	0.662	1.003	0.991	1.015
Is household able to make ends meet (easily) reference category						
Self-perceived health (high: worse)	0.277	0.0029	<0.001	1.319	1.311	1.326
Number of chronic diseases	0.037	0.0020	<0.001	1.038	1.034	1.042
Activities of daily living index (high: has difficulties)	0.078	0.0038	<0.001	1.082	1.074	1.090
Instrumental activities of daily living index (high: has difficulties)	0.073	0.0043	<0.001	1.075	1.066	1.085
Body mass index	−0.003	0.0005	<0.001	0.997	0.996	0.998

Dependent variable: EURO-D. Included cases = 151,354. Poisson loglinear. Model: sex, age, HGS, living with spouse/partner, SRH, Number of chronic diseases, ADL, IADL, BMI, Is household able to make ends meet.

We then calculated the same GEE separately for female and male individuals. In both cohorts, we found that HGS significantly predicted future depressive symptoms (Table 3). However, there is also a low predictive impact associated with it, based on an OR of almost 1. The separate analysis was done to directly detect possible gender differences in HGS, as in the previous GEE (Table 2), male gender showed a significant preventive influence on depression (*B* = −0.169 OR = 0.845 95%CI [0.832, 0.857], *p* < 0.001).

**Table 3:** GEE for female and male individuals.

Male	*B*	Standard error	*p*	OR	95%CI lower	95%CI upper
Constant	0.111	0.0558	0.047	1.117	1.002	1.246
Hand grip strength	−0.005	0.0005	<0.001	0.995	0.994	0.996
Age	−0.003	0.0005	<0.001	0.997	0.996	0.998
Living with spouse/partner (yes)	−0.156	0.0104	<0.001	0.856	0.838	0.873
Is household able to make ends meet (with great difficulties)	0.361	0.0135	<0.001	1.435	1.398	1.474
Is household able to make ends meet (with some difficulties)	0.131	0.0103	<0.001	1.140	1.117	1.163
Is household able to make ends meet (fairly easy)	0.009	0.0096	0.348	1.009	0.990	1.028
Is household able to make ends meet (easily) = reference category						
Self-rated health (high: worse)	0.284	0.0047	<0.001	1.329	1.317	1.341
Number of chronic diseases	0.042	0.0033	<0.001	1.043	1.036	1.050
Activities of daily living index (high: has difficulties)	0.102	0.0065	<0.001	1.107	1.093	1.122
Instrumental activities of daily living index (high: has difficulties)	0.077	0.0070	<0.001	1.080	1.066	1.095
Body mass index	−0.004	0.0010	<0.001	0.996	0.994	0.998

Female	*B*	Standard error	*p*	OR	95%CI lower	95%CI upper
Constant	0.602	0.0384	<0.001	1.826	1.693	1.968
Hand grip strength	−0.011	0.0005	<0.001	0.989	0.988	0.990
Age	−0.006	0.0004	<0.001	0.994	0.994	0.995
Living with spouse/partner (yes)	−0.035	0.0071	<0.001	0.966	0.952	0.979
Is household able to make ends meet (with great difficulties)	0.296	0.0104	<0.001	1.345	1.318	1.373
Is household able to make ends meet (with some difficulties)	0.111	0.0081	<0.001	1.117	1.100	1.135
Is household able to make ends meet (fairly easy)	0.001	0.0077	0.916	1.001	0.986	1.016
Is household able to make ends meet (easily) reference category						
Self-rated health (high: worse)	0.270	0.0037	0.000	1.309	1.300	1.319
Number of chronic diseases	0.035	0.0026	0.000	1.035	1.030	1.041
Activities of daily living index (high: has difficulties)	0.065	0.0047	0.000	1.067	1.057	1.077
Instrumental activities of daily living index (high: has difficulties)	0.069	0.0055	0.000	1.071	1.059	1.083
Body mass index	−0.002	0.0006	<0.001	0.998	0.996	0.999

## 5. Discussion

Our findings confirm an association between low HGS and depressive symptoms. This association was present in cross-sectional as well as in longitudinal analyses.

This association remains stable when controlling for covariates commonly related to depressive symptomology, such as health, economic situation, difficulties in ADL and IADL, sex, and age (56, 57). Many previous studies and meta-analyses have assessed, identified and explained the influence of these variables on depressive symptoms (2, 56, 58–63); however, the aim of the present study was specifically to identify a potential link between HGS and depressive symptoms in community-dwelling adults. While we did identify this link both cross-sectionally and longitudinally, it is worth to note that the association between HGS and depressive symptoms is not strong, as indicated by the standardized beta (wave 4: *β* = 0.019, *p* < 0.001; wave 5: *β* = 0.006, *p* < 0.001; wave 6: *β* = 0.013, *p* < 0.001) or OR [OR = 0.992, 95%CI (0.991, 0.993), *p* < 0.001]. However, the association fits to the existing literature on the relationship between depressive symptoms and HGS (47, 50, 51, 64). Additionally, in our data, this significant association between depressive symptomology and HGS also holds in in female persons. There is a disagreement whether the relationship between depressive symptoms and HGS holds for male as well as female persons, with some studies reporting effects for men only and others finding a relationship in women as well (13, 65). This disagreement regarding the effect of gender as well as the overall relationship between HGS and depressive symptoms (13–15, 18) may be due to various methodological reasons. First, there is considerable heterogeneity in the assessment of HGS. SHARE measured the maximum HGS in contrast to some other studies that used the average HGS (10). Furthermore, the existing studies also differ in which depression score was used and which cofactors were considered (17, 18, 32, 66, 67). In a review of HGS measurements, Roberts and colleagues (10) found various different dynamometers in use, and although the majority of studies utilizes standardized dynamometers with comparable reliability, the procedure of the measurement itself is not standardized. For example, the position of the dynamometer handle plays an important role in the measured strength, and its adjustment is oftentimes not performed correctly. This can lead to an over- or underestimation of strength depending on hand size, and may especially explain the incongruence in gender differences, as women often have smaller hands with longer nails than men and may struggle with inappropriately positioned handles (10). Additionally, the authors report differences in the number of measurements and the time slots between them, which may lead to fatigue effects especially when averaging across results. Likewise, Huang et al. (18) reviewed eight newer studies and also found differences regarding the hands measured (dominant versus bilateral), the participant position (standing, sitting) and the calculation of the final score (maximal or average). In their review, Volaklis et al. (67) report similar inconsistencies with dynamometers of use, handle adjustment, number of measurements and calculation of the final HGS value. Likewise, the assessments of depressive symptomology may vary across studies. Both Volaklis and colleagues (67) and Huang et al. (18) each identified seven different measures of depressive symptoms across eight studies. These scales each contain different items and recommended cut-off scores, leading to an overall incomparability of obtained results. For a comprehensive understanding of the association between HGS and depressive symptoms, a standardized measurement procedure is indispensable to ensure comparability and reproducibility of results. Another possible explanation for the inconsistent gender difference could be that women may have an overall disadvantage in the HGS measurement due to their relatively weaker musculature and potentially lower muscle mass, which could further decline over time. Furthermore, it is recognized that there are gender differences in depression (2).

In addition, our analyses were not limited to single highly selective patient groups, but to community-dwelling older adults. Thus, most participants exhibited healthy HGS and a low level of depressive symptomology. It is probable that the association between a global indicator of health like HGS and depressive symptomology is more pronounced in cohorts with certain diseases than in community-dwelling older adults (7, 18, 65).

For example, in a recent umbrella review regarding the association between depression and mortality, Machado et al. (7) conclude that a strong link was only evident for certain illnesses and specific populations, indicating that the association between depression and physical health is not straight forward and only becomes evident in certain cases (7). The authors interpret these findings with regards to potential mediators between depressive symptoms and health, such as altered health behavior in terms of medication adherence, lifestyle, motivation, and physical inactivity (13, 68–70). In line with this interpretation is the finding in our own analysis that depressive symptoms were closely linked with difficulties in daily activities (iADLs and ADLs) as well as with SRH. Likewise, certain illnesses may also lead to reduced activity and social participation, which is closely linked with depressive symptoms (71).

Another potential mechanism linking HGS and depressive symptoms are common underlying biochemical and inflammatory processes. Muscular strength is influenced by various biological factors, including hormonal balance, inflammation, and oxidative stress. These factors have also been implicated in the development and progression of depression (72, 73). Thus, individuals with lower HGS may have dysregulated physiological processes that contribute to an increased risk of depression (7). Physical fitness, including muscular strength, is known to influence various physiological pathways that are also implicated in depression (61). Regular physical activity and exercise, which contribute to increased muscle strength, have been shown to also modulate neurobiological processes associated with mood regulation (74). Therefore, it is possible that the association between HGS and depression is mediated through shared pathways involving both lifestyle and activity as well as neurochemical systems (7, 30, 73).

Generally, the robust but weak association between HGS and depressive symptoms suggest that this link is more likely to be fueled by common underlying disease processes or motivational deficits. In a recent review, Bohannon (22) summarized a relation between low HGS and a multitude of physical and psychological variables, including but not restricted to depression, cognition, malnutrition, disease status, and falls (22). These findings indicate that despite a relation between depressive symptomology and HGS, this link is not specific to depressive symptoms. While HGS cannot serve as a marker specific to depression, it is indicative of overall poor health, encompassing both physical and mental well-being (22, 75). Thus, when performed appropriately, the HGS measurement could serve as an affordable and quick general screening module for overall health status in order to identify patients at risk that require in-depth assessment. Future research should aim to explore these potential mechanisms in more detail to better understand the underlying pathways linking HGS to categories of depressive symptoms.

## 6. Limitations

Our study is not free of limitations. The data is based on community dwelling people, indicating that the studied population is fairly healthy. For these reasons, only limited statements can be made about people with severe disorders. One of our aims was to assess whether the association between HGS and depressive symptomology that has been found in some previous studies, holds in a large cohort of community-dwelling adults. As expected, in our cohort of community dwelling adults interviewed in a Europe-wide sample, the distribution of low HGS was comparatively low (76), especially when compared to older adults or hospitalized patients (32, 65). While these data match the purpose of our analysis, they do not reflect older or hospitalized persons with certain medical conditions. Thus, our analysis cannot suspend previous research showing a stronger association between HGS and depressive symptoms, as this very association may depend on the studied population and included variables (7, 18). Nonetheless, the fact that we found an association of HGS and depressive symptoms in such community-dwelling adults with low levels of low HGS and depressive symptomology suggests that this effect may be even stronger in sub-cohorts, inviting future research to assess in-depth how the two variables are linked and which mechanisms carry this effect.

Additionally, several variables in the analysis are based on self-reports, including depressive symptoms, SRH, economic situation and daily activities, which means that the answers given cannot be objectified. Issues associated with self-reported data include the fact that people tend to provide socially desirable responses. Additionally, there exists a risk of sampling bias (77). However, there are studies indicating that self-report retains valuable information (78), and all self-report instruments used in the data collection procedure are validated and frequently used. Furthermore, SHARE opted for a standardized CAPI data collection procedure to reduce the risk of response bias in terms of social desirability. Of note, the depressive symptomology described in this analysis is based on self-report and does not replace a professional interview and diagnostic procedure. No conclusions can be drawn about people with severe forms of depressive disorders (79). Additionally, there is only weak agreement at the level of diagnosis of depression between self-rated and clinical diagnosis. Furthermore, there is a tendency to under-report significant depression in a survey of self-rated depressive symptoms (80).

## 7. Conclusion

In conclusion, this study confirms a weak but longitudinally robust association between HGS and depressive symptoms in both male and female community dwelling adults aged 50 years and older. HGS may be indicative of overall poor health including mental and physical aspects. Further standardized research is needed to understand the underlying mechanisms and explore the potential of interventions targeting muscular strength for prevention and management of depressive symptomology as well as improvement of overall health.

## Data availability statement

Publicly available datasets were analyzed in this study. This data can be found at: http://www.share-project.org.

## Ethics statement

The studies involving humans were approved by continuous ethics review by responsible ethics committees (University of Mannheim and Max Planck Society, Germany), as well as national ethics committees in participating countries as part of the SHARE data collection. The studies were conducted in accordance with the local legislation and institutional requirements. The participants provided their written informed consent to participate in this study.

## Author contributions

SM: Formal analysis, Methodology, Writing – original draft. AS: Conceptualization, Formal analysis, Methodology, Writing – review & editing. KGH: Writing – review & editing. TP: Conceptualization, Formal analysis, Methodology, Writing – review & editing.

## Funding

The author(s) declare financial support was received for the research, authorship, and/or publication of this article. SM received funding from a Bundesministerium für Bildung und Forschung (BMBF, Federal Ministry of Education and Research) grant (01GY1804). KGH received funding from the Deutsche Forschungsgemeinschaft (DFG, German Research Foundation) as part of the Clinician Scientist-Program OrganAge, funding number 413668513. TP received funding from a Bundesministerium für Bildung und Forschung (BMBF, Federal Ministry of Education and Research) grant (01GY2301).

## Conflict of interest

The authors declare that the research was conducted in the absence of any commercial or financial relationships that could be construed as a potential conflict of interest.

## Publisher’s note

All claims expressed in this article are solely those of the authors and do not necessarily represent those of their affiliated organizations, or those of the publisher, the editors and the reviewers. Any product that may be evaluated in this article, or claim that may be made by its manufacturer, is not guaranteed or endorsed by the publisher.

## Footnotes

^1^ https://www.share-datadocutool.org/study_units/view/1
