# Supplementary material for: Prospective associations between hand grip strength and subsequent depressive symptoms in men and women aged 50 years and older: insights from the Survey of Health, Aging, and Retirement in Europe
Source: Front Med (Lausanne). 2023 Sep 14;10:1260371. doi: 10.3389/fmed.2023.1260371 (PMC10536140; doi:10.3389/fmed.2023.1260371)
Supplement: Supplementary file 1 [file Table_1.DOCX]

Supplementary Material

**Table 1. Linear regression wave 4 (dependent variable = EURO-D)**

|  | coefficient | p | beta |
| --- | --- | --- | --- |
| constant | 2.591 | <0.001 |  |
| Self-rated health | 0.636 | <0.001 | 0.553 |
| Is household able to make ends meet (with great difficulties) | 0.832 | <0.001 | 0.102 |
| Is household able to make ends meet (with some difficulties) | 0.727 | <0.001 | 0.102 |
| Is household able to make ends meet (fairly easy) | 0.030 | 0.163 | 0.102 |
| Is household able to make ends meet (easily) = reference category | . |  |  |
| Activities of daily living index | 0.750 | <0.001 | 0.093 |
| Instrumental activities of daily living index | 1.223 | <0.001 | 0.078 |
| sex (male) | -0.491 | <0.001 | 0.049 |
| number of chronic diseases | 0.138 | <0.001 | 0.038 |
| age | -0.016 | <0.001 | 0.033 |
| HGS | -0.014 | <0.001 | 0.019 |
| Living with spouse/partner (yes) | -0.207 | <0.001 | 0.013 |
| BMI | -0.021 | <0.001 | 0.013 |

*F*(18, 56064) = 1229.378, p < 0.001, adjusted R^2^ =0.283. Stepwise selection with Akaike information criterion (AIC).

**Table 2. Linear regression wave 5 (dependent variable = EURO-D)**

|  | coefficient | p | beta |
| --- | --- | --- | --- |
| constant | 2.05 | <0.001 |  |
| Self-rated health | 0.631 | <0.001 | 0.564 |
| Is household able to make ends meet (with great difficulties) | 0.968 | <0.001 | 0.126 |
| Is household able to make ends meet (with some difficulties) | 0.342 | <0.001 | 0.126 |
| Is household able to make ends meet (fairly easy) | 0.004 | 0.818 | 0.126 |
| Is household able to make ends meet (easily) = reference category | . |  |  |
| Activities of daily living index | 0.793 | <0.001 | 0.092 |
| Instrumental activities of daily living index | 0.849 | <0.001 | 0.074 |
| sex (male) | -0.536 |  | 0.060 |
| number of chronic diseases | 0.119 | <0.001 | 0.027 |
| age | -0.013 | <0.001 | 0.020 |
| Living with spouse/partner (yes) | -0.209 | <0.001 | 0.014 |
| BMI | -0.019 | <0.001 | 0.011 |
| HGS | -0.008 | <0.001 | 0.006 |

*F*(19, 63779) = 1326.459, p < 0.001, adjusted R^2^ =0.283. Stepwise selection with Akaike information criterion (AIC).

**Table 3. Linear regression wave 6 (dependent variable = EURO-D)**

|  | coefficient | p | beta |
| --- | --- | --- | --- |
| constant | 2.196 | <0.001 |  |
| Self-rated health | 0.645 | <0.001 | 0.569 |
| Is household able to make ends meet (with great difficulties) | 0.941 | <0.001 | 0.146 |
| Is household able to make ends meet (with some difficulties) | 0.227 | <0.001 | 0.146 |
| Is household able to make ends meet (fairly easy) | -0.028 | 0.158 | 0.146 |
| Is household able to make ends meet (easily) = reference category | . |  |  |
| Instrumental activities of daily living index | 0.896 | <0.001 | 0.082 |
| Activities of daily living index | 0.675 | <0.001 | 0.067 |
| sex (male) | -0.479 | <0.001 | 0.046 |
| number of chronic diseases | 0.113 | <0.001 | 0.024 |
| age | -0.013 | <0.001 | 0.020 |
| Living with spouse/partner (yes) | -0.235 | <0.001 | 0.017 |
| HGS | -0.012 | <0.001 | 0.013 |
| BMI | -0.016 | <0.001 | 0.008 |

*F*(14, 64562) = 1704.365, p < 0.001, adjusted R^2^ =0.270. Stepwise selection with Akaike information criterion (AIC).
